# Affinity Purification Mass Spectrometry on the Orbitrap–Astral Mass Spectrometer Enables High-Throughput Protein–Protein Interaction Mapping

**DOI:** 10.1021/acs.jproteome.4c01040

**Published:** 2025-03-03

**Authors:** Lia R. Serrano, Adrian Pelin, Tabiwang N. Arrey, Nicolaie E. Damoc, Alicia L. Richards, Yuan Zhou, Noah M. Lancaster, Trenton M. Peters-Clarke, Anna Pashkova, Gwendolyn M. Jang, Manon Eckhardt, Scott T. Quarmby, Martin Zeller, Daniel Hermanson, Hamish Stewart, Christian Hock, Alexander Makarov, Vlad Zabrouskov, Nevan J. Krogan, Joshua J. Coon, Danielle L. Swaney

**Affiliations:** † Department of Chemistry, University of Wisconsin–Madison, Madison, Wisconsin 53706, United States; ‡ Department of Biomolecular Chemistry, University of Wisconsin–Madison, Madison, Wisconsin 53706, United States; § J. David Gladstone Institutes, San Francisco, California 94158, United States; ∥ Quantitative Biosciences Institute (QBI), University of California, San Francisco, San Francisco, California 94158, United States; ⊥ Department of Cellular and Molecular Pharmacology, University of California San Francisco, San Francisco, California 94158, United States; # Thermo Fisher Scientific GmbH, Bremen 28199, Germany; ¶ National Center for Quantitative Biology of Complex Systems, Madison, Wisconsin 53706, United States; ∇ Morgridge Institute for Research, Madison, Wisconsin 53515, United States; ○ Thermo Fisher Scientific, San Jose, California 95134, United States

**Keywords:** AP-MS, PPIs, Astral, host–pathogen

## Abstract

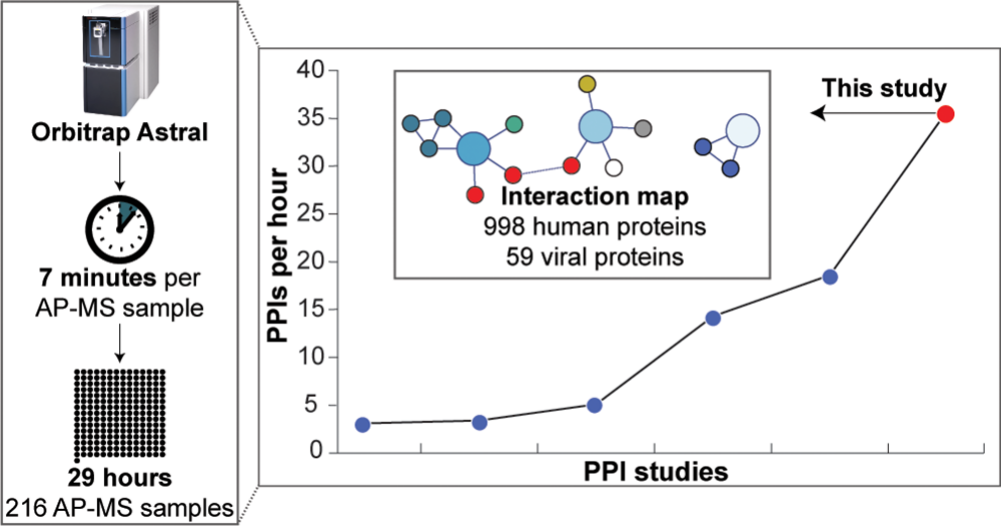

Classical proteomics experiments offer high-throughput protein quantification but lack direct evidence of the spatial organization of the proteome, including protein–protein interaction (PPIs) networks. While affinity purification mass spectrometry (AP-MS) is the method of choice for generating these networks, technological impediments have stymied the throughput of AP-MS sample collection and therefore constrained the rate and scale of experiments that can be performed. Here, we build on advances in mass spectrometry hardware that have rendered high-flow liquid chromatography separations a viable solution for faster throughput quantitative proteomics. We describe our methodology using the Orbitrap–Astral mass spectrometer with 7 min, high-flow separations to analyze 216 AP-MS samples in ∼29 h. We show that the ion-focusing advancements, rapid mass analysis, and sensitive ion detection facilitate narrow-bin data-independent acquisition on a chromatographically practical timescale. Further, we highlight several aspects of state-of-the-art confidence-scoring software that warrant reinvestigation given the analytical characteristics of the Orbitrap–Astral mass spectrometer through comparisons with an enrichment-based thresholding technique. With our data, we generated an interaction map between 998 human proteins and 59 viral proteins. These results hold promise in expediting the throughput of AP-MS experiments, enabling more high-powered PPI studies.

## Introduction

The cellular proteome must be regulated in terms of quantity, time, and spatial organization to perform a diversity of biological functions. While modern high-throughput global proteomics can be used to infer proteome regulation through the measurement of differential protein abundance, these measurements do not reveal proteome organization, including protein localization and protein–protein interactions (PPIs). Affinity purification mass spectrometry (AP-MS) is the gold standard for the detection of PPIs by mass spectrometry.^1−6^ However, its throughput is limited by the need to individually analyze purifications from each protein of interest. The throughput of conventional AP-MS experiments can be influenced by clone availability, cell culture capabilities, sample preparation, and data collection. One way to expedite this workflow is through faster data collection strategies. Typical AP-MS studies often utilize 20–90 min nanoLC–MS/MS data acquisition methods per sample, which are largely driven by the duration of the separation and MS scan speed.^1−3,7,8^ Thus, given the sheer magnitude of the interactome space, higher throughput data acquisition methods are warranted to facilitate the scaling of AP-MS across both a breadth of proteins and a diversity of cellular states.

To increase proteomics throughput, recent studies have merited the use of rapid microflow LC–MS/MS, which requires mass spectrometry instrumentation that is both sensitive enough to compensate for the decreased signal associated with microdroplet ESI and fast enough in scan speeds to measure peptides across the narrower chromatographic peaks produced by microflow gradients.^9−11^ The recently released Thermo Scientific Orbitrap Astral mass spectrometer combines a quadrupole mass filter with both Orbitrap and novel Asymmetric Track Lossless (Astral) analyzers and presents many of the aforementioned requisites to effectively apply fast LC separations for a variety of proteomics applications.

Here, we take advantage of microflow LC–MS/MS and the nearly 200 Hz sampling rate of the Orbitrap–Astral mass spectrometer for high-throughput AP-MS data collection. Our configuration facilitates unprecedented throughput, ultimately enabling us to sample 216 AP-MS samples in ∼29 h. We use this sample set to explore how the existing scoring methods perform with the Orbitrap–Astral data, highlighting several aspects of existing software that can be revisited to account for the enhanced sampling depth. Finally, we implement and provide validation for an enrichment-based thresholding strategy to distinguish 998 confident interactors across 59 unique baits.

## Methods

### Cell Culture and Viral Infection

Vaccinia (VACV) virus protein sequences with a 2× C-term StrepTag were codon-optimized and synthesized by GenScript. These were then cloned into a custom plasmid consisting of the following elements: B14R_left flank region, pox pE/L, codon-optimized Vaccinia bait (variable), pox pE/L, FLuc-P2A-eGFP, and B14R_right. This design permits the use of these plasmids to insert our codon-optimized and tagged bait into the genome of VACV Copenhagen between B14R and B15R. We have this approach to generate individual VACV recombinant viruses expressing 45 baits (i.e., A19L A20R A25L A26L A31R A35R A39R A40R A41L A46R A48R A49R A53R A54L A55R A57R B12R B13R B16R B18R B2R B3R B7R C14L C16L C18L C19L C20L C21L C22L C23L E5R E7R E9L F16L F6L G5R G6R H6R I3L I4L I6L J2R K4L O1L). Recombinant viruses were generated by infecting a 70% confluent six-well U2OS cells with parental VACV Copenhagen at an MOI of 0.01 for 2 h, followed by transfection with 1 μg of shuttle plasmid for 72 h. All viral stocks were subject to WGS NGS for identity validation (correct insertion) and variant screening of the VACV genome. To generate AP-MS samples, confluent A549 cells were infected with recombinant VACV at an MOI of 1 for 24 h, after which cells were harvested using 5% EDTA and stored as cell pellets at −80 °C until further processing.

For 22 baits, recombinant VACV either failed to rescue or were at a high risk of doing so due to their housekeeping functions (i.e., A14.5L A18R A22R A23R A24R A29L A2L A50R A5R A7L A8R C9L D4R D5R F10L H1L H3L L3L VACWR002 VACWR003 VACWR005 VACWR012). To generate AP-MS samples for these baits, confluent A549 cells were infected with parental VACV Copenhagen at an MOI of 10 for 2 h, followed by transfection with 15 μg of custom plasmid (described in the previous paragraph) for 24 h, after which cells were harvested using 5% EDTA and stored as cell pellets at −80 °C until further processing. Copenhagen strain VACV was a generous gift from Dr. Grant McFadden (Arizona State University, US).

### Affinity Purification

Frozen vaccinia virus-infected A549 cell pellets were thawed on ice for 15–20 min. The thawed cells were suspended in 1 mL of Lysis Buffer [IP Buffer (50 mM Tris-HCl, pH 7.4 at 4 °C, 150 mM NaCl, 1 mM EDTA) supplemented with 0.5% Nonidet P 40 Substitute (NP40; Fluka Analytical) and cOmplete mini EDTA-free protease and PhosSTOP phosphatase inhibitor cocktails (Roche)]. Samples were then frozen on dry ice for 10–20 min and partially thawed at 37 °C before incubation on a tube rotator for 30 min at 4 °C and centrifugation at 13,000*g*, 4 °C for 15 min to pellet debris. After arraying lysates into a 96-well Deepwell plate and removing an aliquot, the plate was kept in ice for affinity purification on the KingFisher Flex Purification System (Thermo Scientific) as follows: MagStrep “type3” beads (30 μL; IBA Lifesciences) were equilibrated twice with 1 mL of wash buffer (IP buffer supplemented with 0.05% NP40) and incubated with ∼0.95 mL of lysate for 2 h. Beads were washed three times with 1 mL of wash buffer and then once with 1 mL of IP buffer. The beads were released into 75 μL of denaturation–reduction buffer (2 M urea, 50 mM Tris-HCl pH 8.0, 1 mM DTT) dispensed into a 96-well KFF microtiter plate for sample processing (described below). The KingFisher Flex Purification System was placed in a cold room and allowed to equilibrate to 4 °C overnight before use. All automated protocol steps were performed using the slow mix speed and the following mix times: 30 s for equilibration/wash steps, 2 h for binding, and 1 min for final bead release. Three 10 s bead collection times were used between all steps.

### On-Bead Digestion

Bead-bound proteins were denatured and reduced at 37 °C for 30 min, and after being brought to room temperature, alkylated in the dark with 3 mM iodoacetamide for 45 min and quenched with 3 mM DTT for 10 min. Proteins were then incubated at 37 °C, initially for 4 h with 1.5 μL trypsin (0.5 μg/μL; Promega) and then another 1–2 h with 0.5 μL additional trypsin. To offset evaporation, 15 μL of 50 mM Tris-HCl, pH 8.0 was added before trypsin digestion. All steps were performed with constant shaking at 1100 rpm on a ThermoMixer C incubator. The resulting peptides were combined with 50 μL of 50 mM Tris-HCl, pH 8.0 used to rinse beads and acidified with trifluoroacetic acid (0.5% final, pH < 2.0). The acidified peptides were desalted for MS analysis using a BioPureSPE Mini 96-Well Plate (20 mg PROTO 300 C18; The Nest Group, Inc.) according to standard protocols.

### LC–MS/MS Analysis

Dried tryptic digests of affinity-purified baits and the respective protein interactors were resuspended in 5% acetonitrile and 0.1% formic acid in water. Each sample was injected from a 96-well plate and loaded onto 15 cm EASY-Spray PepMap RSLC columns (150 μm × 150 mm i.d. × o.d.) packed with 2 μm C18 particles (Thermo Fisher) and electrosprayed into an Orbitrap–Astral mass spectrometer. Reversed-phase chromatographic separations were performed using a Vanquish Neo UHPLC System using 0.1% formic acid in water for mobile phase A and 80% ACN and 0.1% formic acid in water for mobile phase B at a flow rate of 2.5 μL/min. Mobile phase B increased from 4 to 8% across 0.2 min, 8 to 20% from 0.3 to 4.0 min, 20 to 35% until 5.8 min, 35 to 99% until 6.2 min, and then held at 99% for 30 s before the column was washed and re-equilibrated with 100% mobile phase A. Electrospray ionization was achieved using a spray voltage of 1.9 kV on the Thermo Scientific EASY-Spray Source. The ion transfer tube was maintained at 290 °C, and the ion funnel RF was set to 40%. MS1 scans were collected in the Orbitrap every 0.6 s (∼every 122 scans) using the 240,000 resolving power setting. Ions were injected for 5 ms or until a 1e6 AGC target was attained for MS1 scans. Two Th windows were isolated across 380–980 *m*/*z* using the quadrupole for MS2 analysis, requiring an injection time of 3 ms or until an AGC target of 5e4 was met. Precursors were fragmented using 25% NCE, and fragments ranging from 150 to 2000 were measured using the Astral mass analyzer.

### Data Analysis

Thermo RAW files were searched using Spectronaut 18 directDIA+ (Deep). For the initial database search used to generate a predicted DIA library, the files were searched with the Pulsar engine against a FASTA file containing 103,859 human protein sequences including isoforms from the Swiss-Prot database (reviewed) and TrEMBL database (unreviewed) downloaded on 4/19/2023 concatenated to 210 Vaccinia virus protein sequences. Tryptic peptides between seven and 52 amino acids with at most two missed cleavages were considered. Carbamidomethylated cysteine was set as a fixed modification, and acetylated protein N-term and oxidized methionine were set as variable modifications—allowing up to five per peptide. The first search to determine the systematic mass error was set to 40 ppm before a narrower dynamic search tolerance was implemented for peptides to be included in the directDIA library. For the predicted library search, the decoy generation method was set to “Mutated”. Protein identifications were filtered to meet a 1% protein-level false discovery rate (FDR) threshold, and the IDPicker^12^ algorithm was used for protein inference. All other settings not listed here were set to default Spectronaut settings. Baits with triplicates that were calculated to have Pearson correlations below 0.62 were excluded from downstream analysis (Supporting Information Figure S2). PPIs were scored using SAINTq (version 0.0.4) and MiST software.^13,14^ Peptide-level intensity information was used for scoring for SAINTq, and protein-level intensity information was used for MiST scoring. Additionally, all interactions were required to have a minimum of three detected intensity values (i.e., three nonimputed values).

Complementary PPI confidence scoring analyses were performed according to the method described by Keilhauer et al.^15^ with minor modifications. Missing values were imputed by randomly sampling values from a modeled normal distribution, simulating the mass spectrometry noise. This distribution was defined as 1.8 standard deviations from the mean of the distributions of the log_2_-transformed protein quantification values. The width of the noise distribution was set to equal 0.3 of the standard deviation of the distribution of the log_2_-transformed protein quantification values. Points from this distribution were selected using the choice function from the built-in Python module, randomly. Protein fold-changes were then calculated between each bait relative to the averaged intensity of that protein across all other samples in the experiment. Two-sided *t* tests were performed using the “ttest_ind” function from the SciPy python package (version 1.10.0) stats module using default settings. *P*-values describing the significance of each fold-change were corrected using the Benjamini–Hochberg method for multiple-hypothesis testing.^16^ This *p*-value correction was performed using the “multi” function from the Python library statsmodels (v. 0.13.5) module, “stats”. Bait-specific thresholding was then applied to each experiment using the following algorithm: all preys with *q*-values greater than the function described in eq 1 were considered weak interactors, while those greater than the function described in eq 2 were considered strong interactors.
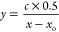
1
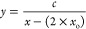
2

In eqs 1 and 2, *x*_o_ denotes the standard deviation of the protein intensities quantified from a specific bait, while *c* was optimized by sweeping through values 0 to 70 in increments of one. The *c* term corresponding to the elbow of the resulting curve plotting true positive rate versus *c* was used for thresholding. The elbow in this case is defined as the point on the true positive rate versus *c* curve that has the farthest distance from the line connecting the first and last points of the curve. True positives were designated as preys that exhibited a Pearson correlation (“corrcoef” function from NumPy v. 1.23.5) of at least 0.4 with 95% of copurified preys. This correlation value was established through observation of the point at which the protein–protein correlation values deviated from a normality. The final value for c applied to all bait thresholds was 31. The final list of interactors for each bait included all proteins meeting the “weak interactor” threshold. Additionally, all interactions were required to have a minimum of three detected intensity values (i.e., three nonimputed values).

The STRING physical links database used for validation was downloaded on 8/24/2024 (9606.protein.physical.links.v12.0). CORUM complex data were downloaded on 5/7/2023 from the Ma’ayan Laboratory of Computational Systems Biology database Harmonizome 3.0 (gene_attribute_edges).^17^ CORUM complex enrichment was determined using the “fisher_exact” function from the SciPy Python package module “stats”.

## Results and Discussion

### Rationale for Fast Gradients Coupled to Orbitrap–Astral MS

**Figure 1:**
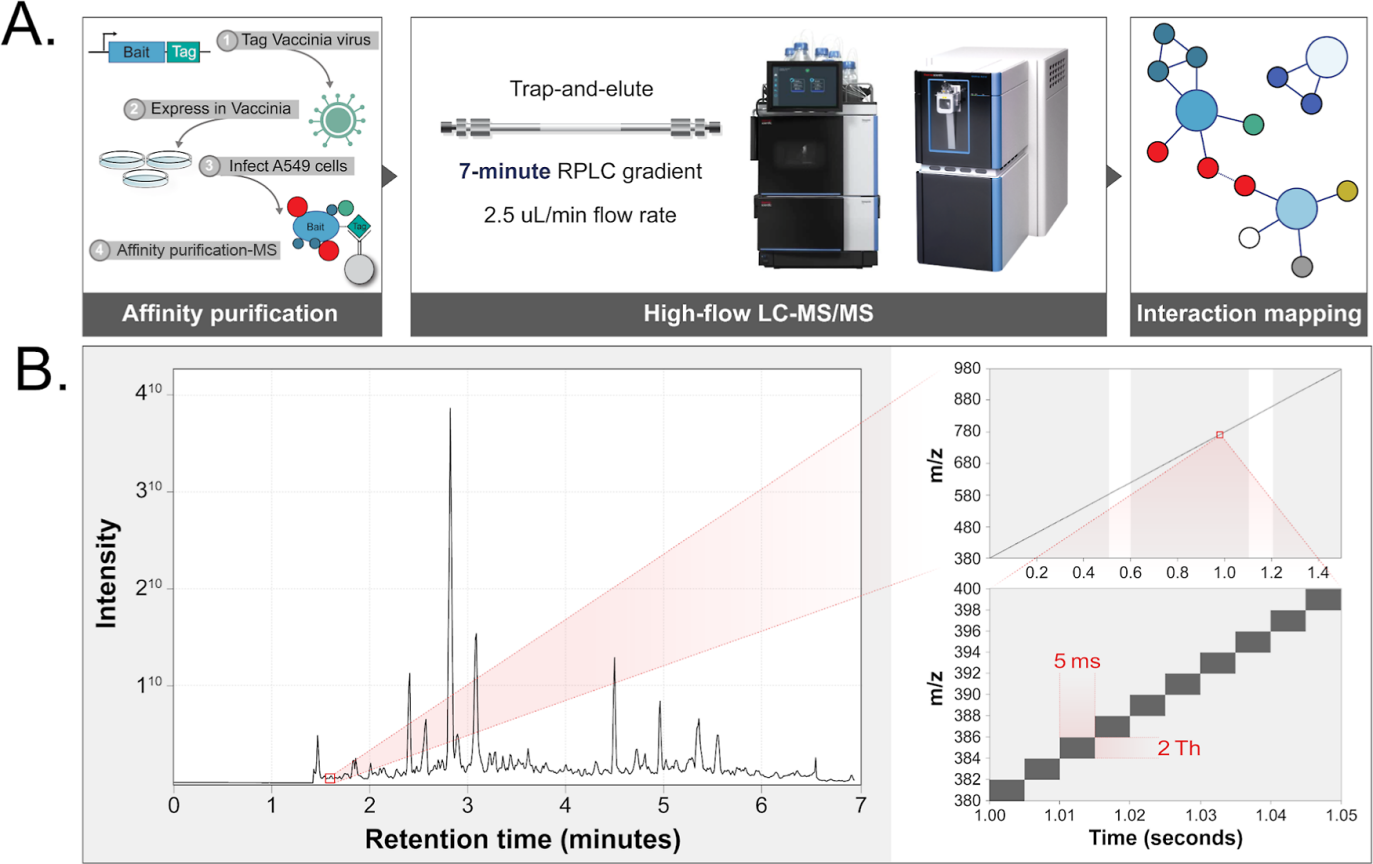
Analytical workflow for fast gradients coupled to Orbitrap–Astral MS for AP-MS experiments. (A) Analytical workflow for high-throughput LC–MS/MS analysis of Vaccinia viral protein-purified human proteins. (B) Over a 7 min active gradient, MS1 scans were collected in the Orbitrap every 0.6 s (∼every 122 scans) using 240,000 resolving power setting. Two Th windows were isolated across 380–980 *m*/*z* for MS/MS analysis in the Astral mass analyzer.

**Figure 2:**
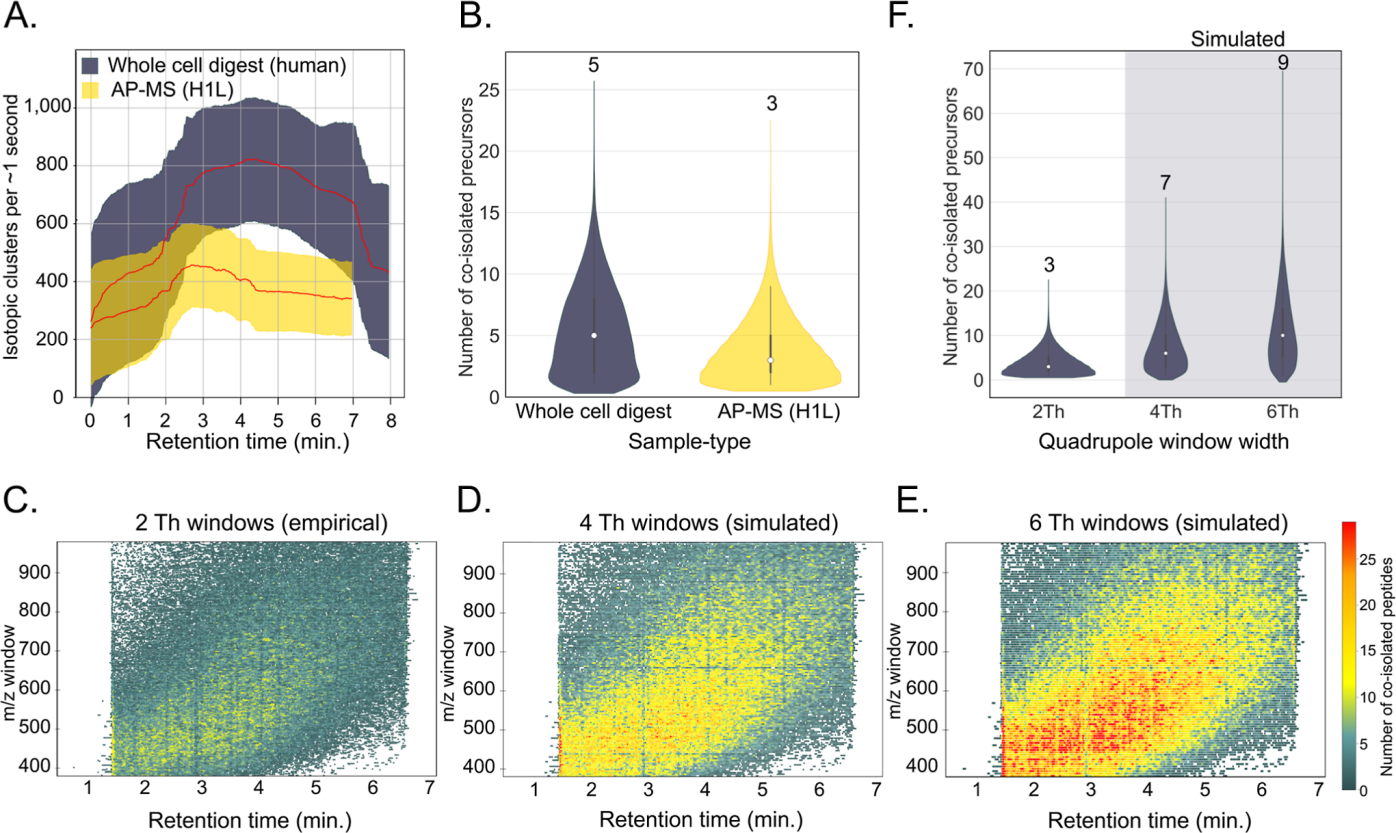
Assessment of fast gradients interfacing with narrow-window DIA on the Orbitrap–Astral MS system. (A) Number of peptidic isotopic clusters detected in Orbitrap MS1 events were counted and plotted as a function of retention time for the H1L-purified interactors, showing a median elution rate of 392 peptidic features per ∼1 s (*n* = 130,464 isotopic clusters) and a whole HAP1 proteome digest separated with a 7 min gradient and analyzed on the Orbitrap–Astral MS system, resulting in a rate of 706 isotopic clusters per second (*n* = 219,039 isotopic clusters). (B) Distribution of the number of coisolated precursors for the whole HAP1 proteome digest and H1L-purified A549 proteins show medians of 5 and 3, respectively. (C–E) Number of coisolated peptides in each (C) 2, (D) 4, and (E) 6 Th window is represented as heat. Note that two coeluting peptides are defined as any overlap between baseline-to-baseline elution profiles, and 4 and 6 Th DIA windows were simulated for the empirically measured precursors. (F) Distributions of the number of coisolated precursors for the empirical 2 Th windows, as well as the simulated 4 and 6 Th DIA windows. The median value is represented above each distribution.

Technological impediments have stymied the throughput of AP-MS sample collection, resulting in bottlenecks in both the rate and scale of the experiments that can be performed. Given the myriad of configurations in which a proteome can organize, there is a technological gap that must be addressed. We explore how the enhanced analytical capabilities of Orbitrap–Astral MS, enabled by key architectural innovations, can possibly expedite AP-MS experiments through its compatibility with 7 min, high-flow separations. To demonstrate the practicality of this analytical format for AP-MS, we first evaluated how effectively the narrow isolation windows enabled by Orbitrap–Astral MS could interface with fast microflow gradients using a single AP-MS sample as an example. Affinity purification was then performed to enrich H1L-interacting proteins from Vaccinia virus-infected A549 cells (Figure 1A), and the resulting samples were analyzed on an Orbitrap–Astral MS system using an accelerated LC gradient (7 min active gradient, 8 min total MS acquisition time) in a narrow-window (2 Th) DIA method (Figure 1B). We compared the spectral complexity of this AP-MS sample to that of a whole cell lysate from HAP1 cells. Both samples were separated with a 7 min active gradient using 2 Th DIA windows (300 windows in total).^18^ The highly complex whole cell lysate resulted in the Orbitrap MS1 detection of 706 isotopic clusters per ∼1 s, while the reduced complexity of the AP-MS sample resulted in the detection of 392 isotopic clusters per second (Figure 2A).

Given these characteristics of the chromatographic separations, we investigated how effectively the narrow DIA windows enabled by the ∼195 Hz sampling rate purified the coeluting peptides. To this end, we plotted the center mass versus retention time of the DIA scan, setting the heat of the data point to be proportional to the number of coisolated peptides. Thus, for two peptides to be considered coisolated, they would need to have any point of their respective baseline-to-baseline elution peak overlapping and a DIA window isolation that encompasses both mass-to-charge ratios. We find that our 2 Th DIA windows result in a median of only three coisolated peptides per MS2 scan for the AP-MS sample and a median of five coisolated peptides for the HAP1 digest (Figure 2B,C and Supporting Information Figure S1). Thus, our analysis demonstrates that the combination of reduced sample complexity and the rapid cycle time of Orbitrap–Astral MS, even with narrow DIA windows, mitigates the impact of increased peptide coelution inherent to faster gradients on selectivity. Lastly, for the AP-MS sample, we also simulated the impact of larger DIA windows (4 or 6 Th) on peptide coisolation, as this would have the benefit of reduced cycle time; however, doing so increased the median number of coisolated peptides to seven and nine, respectively (Figure 2C–F). As these larger DIA windows exceeded the coisolation complexity observed in our whole cell lysate, we elected to utilize the 2 Th window for all subsequent experiments.

### Benchmarking Rapid Separations for Label-Free Quantification

To assess the performance of this method more broadly, we then expanded our analysis to a larger set of 216 AP-MS samples, comprising 67 unique Strep-tagged Vaccinia protein baits in biological triplicate (except for two baits with 6 replicates) as well as nine mCherry negative controls. Each sample was loaded onto the reversed-phase column, chromatographically separated at 2.5 μL/min across a 7-min active gradient, and electrospray-ionized into the Orbitrap–Astral mass spectrometer for analysis. Raw files were then searched using directDIA on Spectronaut and filtered to 1% protein FDR. Eight baits that did not meet the quality control criteria (Methods) were excluded from further analysis, resulting in 59 remaining baits in triplicate (except for C20L, C14L, G5R, and C23L in duplicate).

**Figure 3:**
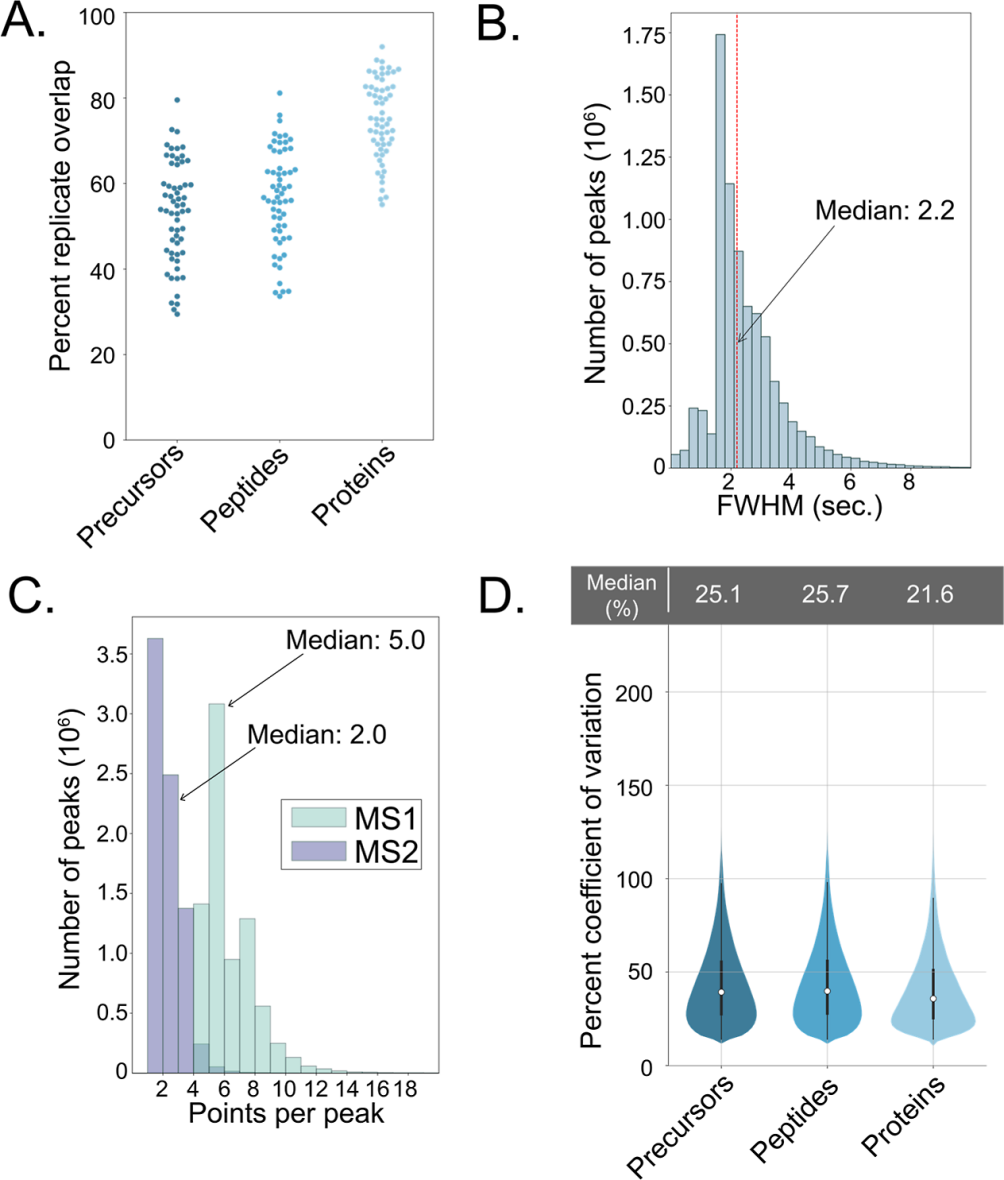
Quantification benchmarking. (A) Percent of shared protein, peptide, and precursor identifications across biological replicates. (B) Distribution of the full width at half-maximum peak height for precursors identified from all samples. (C) Number of points per MS2 peak and MS1 peak across all samples. (D) Percent CV of protein and peptide quantities across all samples.

Our method was highly sensitive in both protein and peptide detection, resulting in an average of 39,541 precursors, 31,508 peptides, and 4000 proteins across all samples. Such detections were also reproducible with a high degree of overlap in protein and peptide detection across replicates (Figure 3A). Beyond protein detections, we next sought to evaluate how the pairing of our rapid LC–MS gradient and ∼195 Hz sampling rate impacted various metrics of quantitative performance. Our DIA method performed an Orbitrap MS1 scan every 0.6 s and cycled through the entire DIA range (300 Astral MS/MS scans) every ∼1.5 s. Over the course of an average chromatographic peak width of 2.2 s (Figure 3B), this resulted in a median of five points per peak at the MS1 level and two points per peak at the MS2 level (Figure 3C). While this sampling rate is insufficient for accurate quantification at the MS2 level, it did enable reproducible quantification at the MS1 level,^19,20^ with a median coefficient of variation (CV) across replicates of 25.7% and 21.6% for peptides and proteins, respectively (Figure 3D). These values are comparable to those derived from AP-MS experiments using longer gradients (18–30% CV).^21,22^

### PPI Scoring Software Evaluation

**Figure 4:**
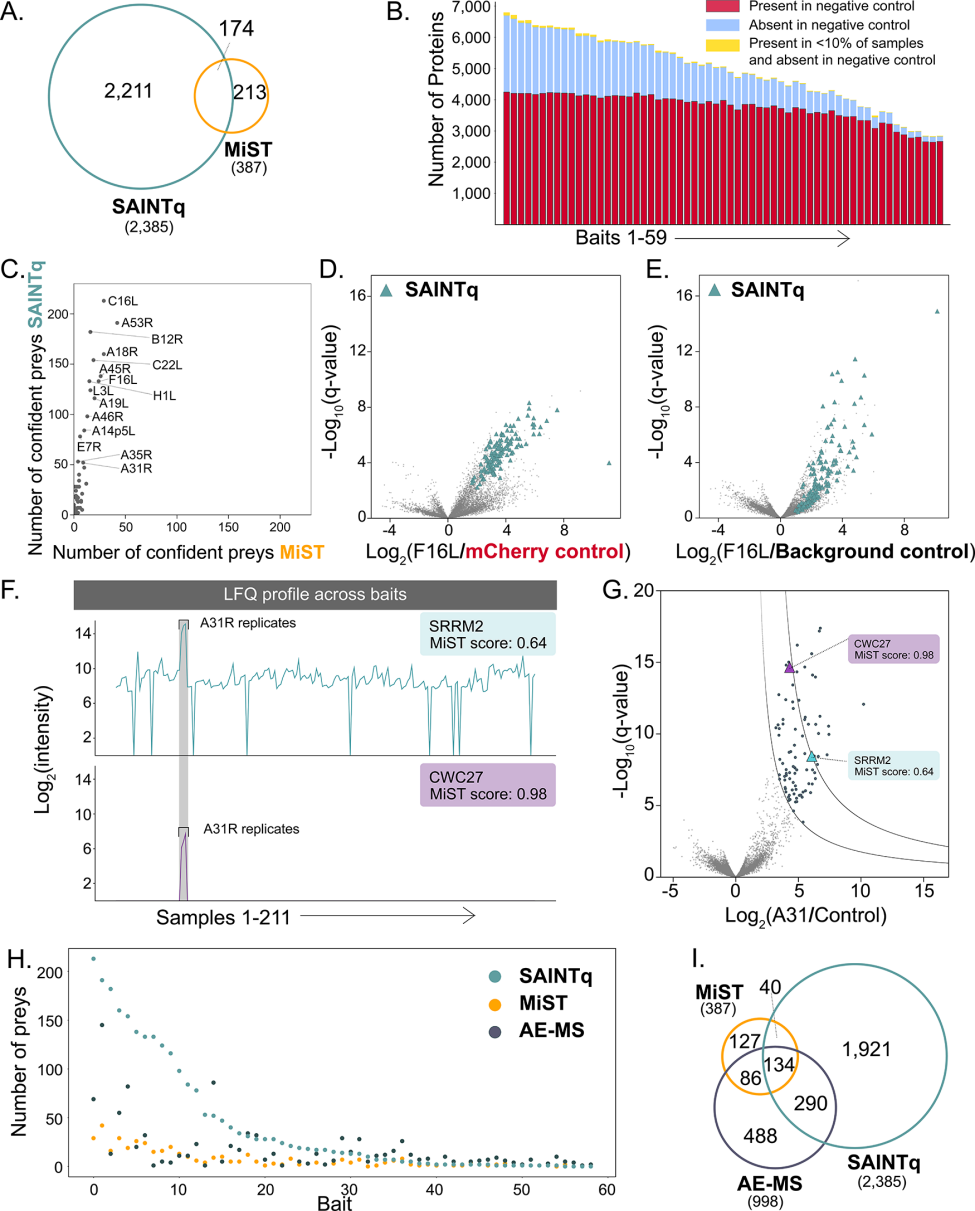
Characterization and investigation of scoring software discrepancies. (A) Overlap in high-confidence PPIs between SAINTq and MiST. (B) Number of proteins quantified across biological replicates of each sample color-coded by the presence (red) in the control sample, absence (light blue) in the control sample, or presence in less than 10% of all baits and absence in the control sample (yellow). 4366 proteins were quantified in the nine control samples, and they were present in 62–97% of the noncontrol samples, whereas the percentage for proteins not present in the control samples is 3–37%. (C) Number of confident preys per bait returned by SAINTq vs MiST. (D) Comparison of *q*-value and log_2_(fold-change) space when using the mCherry controls or (E) quantified proteins across all experiments to model the “background proteome”. For each protein, *t* tests were used to calculate *p*-values describing the difference in the mean protein intensity across three biological replicates. *P*-values were corrected using the Benjamini–Hochberg approach for multiple hypothesis testing. Teal triangles indicates proteins that meet SAINTq threshold requirements for “confident interactors”, respectively. (F) Log_2_-transformed intensity of a low-MiST-scoring (0.64) prey SRRM2 and high-MiST-scoring (0.98) prey CWC27 for the A31R bait pull-downs across all samples. (G) AE-MS thresholding applied to the A31R pull-down. Inner curve indicates the minimum threshold for weak interactions, while the outer curve indicates the minimum threshold for strong interactions. The low-MiST-scoring (0.64) prey SRRM2 and high-MiST-scoring (0.98) prey CWC27 are shown as blue and purple triangles, respectively. (H) Number of preys per bait are shown for MiST (orange), AE-MS (dark blue), and SAINTq (teal). (I) Overlap of assessed software MiST and SAINTq confident interactors with the AE-MS thresholding method.

We next proceeded to analyze our data to determine high-confidence PPIs using two well-established algorithms that are designed to utilize intensity data, as opposed to spectral counts, and have been employed in previous experiments studying host–pathogen PPIs. Importantly, while both algorithms evaluate the abundance and reproducibility of a given protein, they take highly complementary approaches in utilizing protein specificity to determine the PPI confidence. In the case of SAINTq, protein intensities are compared for each bait independently to the intensity distribution of the negative control samples using a semisupervised mixture model to assess the probability of an interaction being true, allowing for FDR calculations. Here, we used DIA peptide quantification data^23,24^ as the input, designated the nine mCherry pull-downs as negative controls, and used a BFDR ≤ 0.05 as our cutoff for high-confidence PPIs. In contrast, MiST^14^ compares each bait against all other baits and prioritizes proteins with high specificity to a single bait. Here, we again used DIA peptide quantification data as the input and required a MiST score cutoff of at least 0.7.^21^ This analysis returned 2385 PPIs crossing our threshold with a median of nine (average of 40) PPI per bait for SAINTq and a total of 387 total interactions with a median of four (average of 7.3) PPIs per bait for MiST (Figure 4A). We observed that the large discrepancy in the number of high-confidence PPIs between SAINTq and MiST was driven by a few baits with an outsized number of detected proteins relative to the negative control samples (Figure 4B,C). Specifically, we found that while there was a substantial number of proteins detected in the mCherry negative controls (4366 proteins), there were yet other 3141 proteins that were exclusively detected across the Vaccinia virus protein AP-MS samples. This result highlights an important assumption of the SAINTq algorithm, in which the negative controls are expected to be highly representative of the background binding to the beads for all AP-MS baits, which is not uniformly true in large AP-MS studies. Several publications have considered proper “background” proteome modeling for AP-MS experiments, highlighting the various biases of experimentally derived negative controls.^7,13^ To increase the size of our control group and get a more comprehensive background model—which has been cited to improve the significance of enriched and true interactors^15^—we tested the effect of all other baits as controls for intensity-based enrichment calculations as opposed to just using the protein intensities from the mCherry control (Figure 4D,E). When increasing the size of the control group, we see that many prey are shifted left, indicating that while these proteins are more abundant in the F16L samples as compared to mCherry, they are commonly observed as background proteins across the entire data set. Overall, we suspect that the greater sensitivity and resultant deeper sampling achieved with the Orbitrap–Astral MS could contribute to the greater protein detection seen in several baits. This effect suggests that the current SAINTq semisupervised model may require updating of FDR calculations to accommodate these deeper proteomic data sets. Further, we show that increasing the size of our control group results in greater distinction of prey from the other proteins in the sample, suggesting that the large number of detected proteins renders our experimental controls unfit for controlling false positives. In contrast, the average number of PPIs per bait for MiST was below what is commonly observed, particularly in light of the large number of proteins detected in each sample (Figure 4B). We hypothesized this could be the result of the enhanced sensitivity of Orbitrap–Astral DIA data, in that there may be a greater number of instances where low-intensity protein signals are detectable, resulting in data sets with less bait-exclusivity in protein detection. This is critical, as protein specificity is the most highly weighted parameter in the MiST algorithm. For example, for the Vaccinia protein A31R, we found the minor spliceosome protein CWC27 to be exclusively detected with this bait, resulting in a high MiST score (0.98), passing our ≥0.7 threshold. In contrast, the SRRM2 minor spliceosome protein was likewise found to be highly enriched in abundance for A31R; however, it is also found to be a common background protein, and this lack of specificity results in a MiST score (0.64) below the cutoff for high-confidence PPIs (Figure 4F).

Based upon these observations, we instead chose to utilize the affinity enrichment mass spectrometry (AE-MS) PPI scoring paradigm introduced by Keilhauer et al. (Methods), which applies a bait-specific, enrichment-based threshold that has been demonstrated to rescue weaker interactions.^15^ As with MiST, this method utilizes the large control group, i.e., every other bait in the experiment, thus avoiding a strong dependency on a small set of negative controls to define true interactors. However, unlike the weighted parameters for abundance, reproducibility, and specificity of the MiST algorithm, the AE-MS scoring approach performs a statistical significance test of the bait-specific enrichment and defines sample-specific *q*-value thresholds, distinguishing both weak interactors (inner curve) and strong interactors (outer curve). This approach is thus more sensitive to the detection of proteins, such as SRRM2, which are commonly detected as background proteins. In revisiting the example in Figure 4F, we observed that AE-MS PPI scoring results in both SRRM2 and CWC27 now passing the thresholds for interactors of this bait (Figure 4G). More broadly, we found this scoring approach resulted in the detection of 998 PPIs across our entire data set, with a median of eight (average of 16.9) PPIs per bait (Figure 4H). Of these interactions, we find a substantial fraction overlap with PPIs also identified by SAINTq or MiST (Figure 4I and Supporting Information Data Table). Importantly, baits with inflated PPI identifications have fewer confident PPIs compared to SAINTq, while many interactions emerged from samples that did not produce any confident interactions meeting the criteria of the other software (Figure 4H).

### Cross-Validation of Interaction Scoring with External Databases

**Figure 5:**
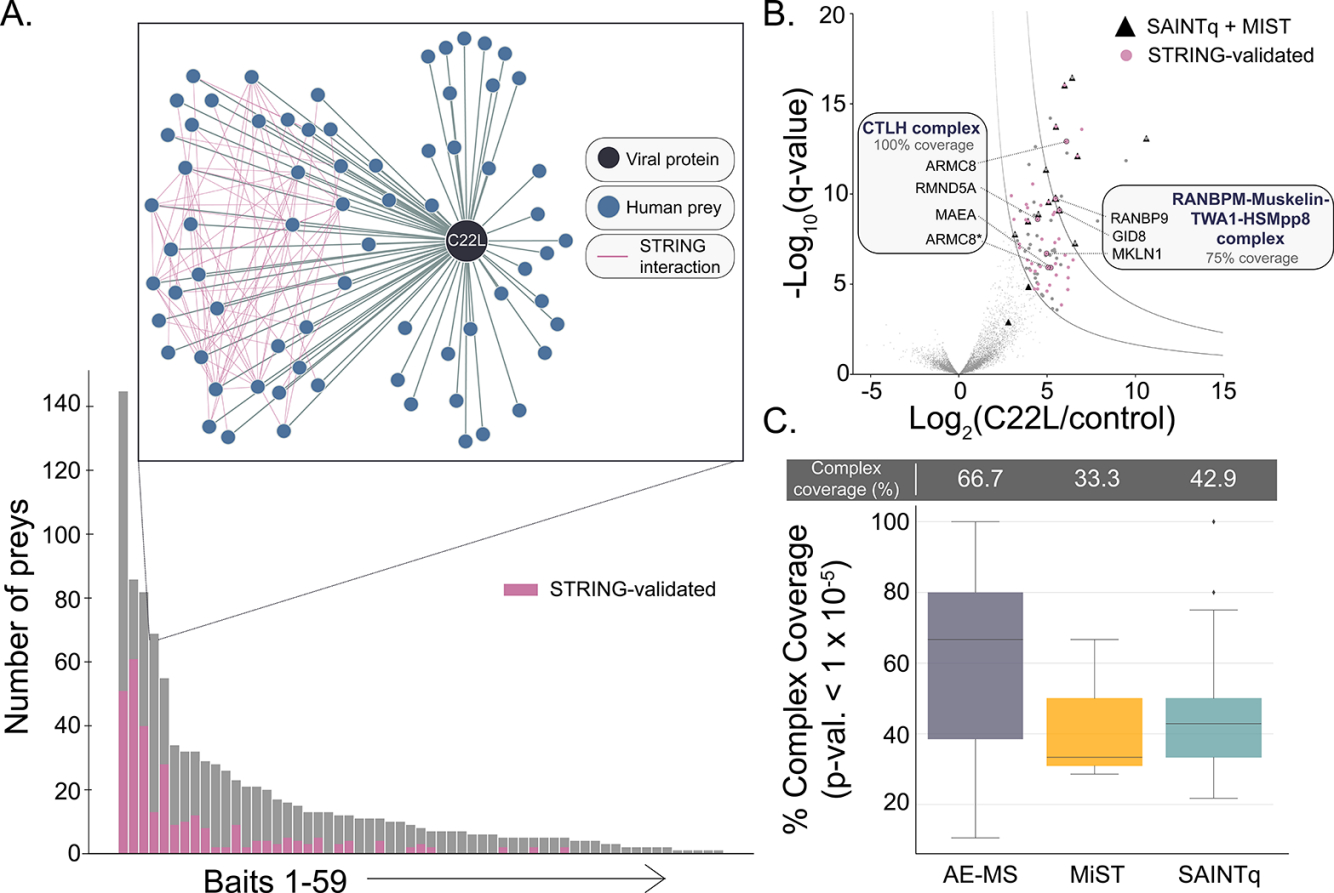
Validation of AE-MS thresholding on rapid Orbitrap–Astral data. (A) AE-MS preys across baits. Pink indicates preys that have at least one cited STRING interaction with a score ≥0.5 with another copurified prey. Example of network graph depiction of C22L pull-down, showing edges (pink) between STRING interactions with a score ≥0.5 from the physical links database. (B) AE-MS thresholding applied to C22L with several recovered CORUM complex examples. Black trianges indicate preys that were also found to pass thresholds in using both SAINTq and MIST. (C) Comparison of CORUM complex enrichment in confident interactors derived from AE-MS thresholding, SAINTq, and MiST. Enrichment *p*-values of CORUM complexes within the set of confident interactors for each bait were calculated using Fisher’s Exact Test. The distributions of the percent complex coverage for all complexes with *p*-values <1 × 10^–5^ are plotted for each method. Only complexes with at least two participants in a given sample were considered for analysis. Only proteins that were detected in the experiment were considered in the calculation for percent coverage.

To validate the preys for each bait returned by the modified AE-MS method, we compared them to cited interactions from the STRING physical links database.^25^ We considered a prey “STRING-validated” if there was at least one other copurified prey in that enrichment with an interaction score of 0.5 in STRING (Figure 5A). Further, we highlight several examples where the modified AE-MS thresholding recovers CORUM complex members as “confident interactors” that do not meet SAINTq and MiST acceptance criteria (Figure 5B).^26^ For example, the MKLN1 protein from the RANBPM–Muskelin–TWA1–HSMpp8 complex (75% complex coverage) in the C22L pull-down is recovered with the AE-MS scoring approach. The overall gain in CORUM complex coverage is summarized in Figure 5C, demonstrating a greater enrichment of CORUM complexes with highly significant *p*-values (<1 × 10^–5^) in the modified AE-MS method set as compared to the SAINTq- and MiST-derived set of confident interactors.^15^ This is particularly notable considering the high number of SAINTq-derived interactors relative to those returned by the AE-MS thresholding method, suggesting that this validation strategy is robust to inflated numbers of proteins. Because of these results, we further analyzed the set of interactions returned by the AE-MS approach for potential biological insights. Figure 5A and Supporting Information Figure S3 show graph depictions of the resultant PPI network. Edges between human proteins were derived from STRING^25^. Altogether, we conclude that the AE-MS method is a valuable approach to analyzing the Orbitrap–Astral-derived DIA data.

## Conclusions

PPI mapping captures a higher dimension of information than whole-proteome LC–MS/MS analysis. AP-MS experiments query one protein of interest in one cellular context per run—creating an obvious throughput bottleneck for systems-level analyses. We demonstrate here that high-flow, rapid LC–MS/MS acquisition is capable of generating high-fidelity protein interaction networks with the Orbitrap–Astral MS system. Our 7 min method achieved sufficient depth and reproducibility to generate an interaction map consisting of 998 confident virus–host or virus–virus protein interactions (with a median of eight PPIs per bait) winnowed down from 328,461 unfiltered interactions measured across all samples. While this rapid acquisition speed provided sufficient quantitative precision for discovery-based APMS experiments, future work may prioritize longer gradients or faster cycle times to increase the points per peak detected and consequently the quantitative precision and accuracy achieved.^19^ We highlight the strengths and weaknesses of standard PPI scoring approaches when applied to the Orbitrap–Astral DIA data. In particular, paradigms that determine scores on the basis of weights optimized using mass spectrometry data from less sensitive instruments, or quantification achieved by spectral counting, may underestimate PPIs. Further, we highlight with our data that enhanced sampling depths can threaten false-positive control when proper modeling of the background binders in algorithms is not attained. We find that an AE-MS analysis as described by Keilhauer et al.^15^ can balance these sensitivity versus specificity considerations when analyzing the highly comprehensive Orbitrap–Astral DIA data and support this with cross-validation by external protein interaction/complex databases such as STRING and CORUM.

As we push technological advancement, it is important to reconsider former data analysis paradigms that coevolve with the instrumentation used to sample biological phenomena. Here, we highlight several aspects of existing PPI scoring software that warrant revalidation for data generated with Orbitrap–Astral MS, which has demonstrated enhanced analytical performance in metrics such as sensitivity, speed, and depth. Nevertheless, the data reported here demonstrate that high-flow, rapid chromatographic separations provide sufficient depth and reproducibility when coupled with the Orbitrap–Astral MS for quality, information-rich, AP-MS-based PPI experiments. Crucially, this data set was achieved in only 29 h, symbolizing high potential to more efficiently and thoroughly sample the vast interactome space. A powerful implication of this throughput is that the the reanalysis of the cumulative BioPlex 3.0 sample set, which originally required ∼1460 days of active LC–MS time, could be completed in only ∼166 days—representing more than an 8-fold increase in data collection throughput (Supporting Information Figure S4).^1−3,8,10,21,27^

## Data Availability

The raw data for the proteomics data sets in this study have been deposited to the MassIVE database under the accession number MSV000096304.

## Supporting Information Available

AEMS_unfiltered: all interactions and AE-MS-based scoring metrics; SAINTq_unfiltered: all interactions and SAINTq-based scoring metrics; MiST_unfiltered: all interactions and MiST-based scoring metrics; AEMS_confident: confident interactions derived from AE-MS scoring; SAINTq_confident: confident interactions derived from SAINTq scoring; and MiST_confident: confident interactions derived from MiST scoring (XLSX)
Co-isolation analysis for HAP1-cell lysate digest separated with a 7-min active gradient and analyzed with Orbitrap–Astral MS (2 Th DIA window method); Pearson correlation coefficients for all replicates of analyzed baits; bait interactions in a network graph depiction for PPIs returned by the AE-MS thresholding method; and throughput comparisons between this study and selected PPI mapping studies (PDF)
