# Supplementary material for: Affinity Purification Mass Spectrometry on the Orbitrap–Astral Mass Spectrometer Enables High-Throughput Protein–Protein Interaction Mapping
Source: J Proteome Res. 2025 Mar 3;24(4):2006–16. doi: 10.1021/acs.jproteome.4c01040 (PMC11976844; doi:10.1021/acs.jproteome.4c01040)
Supplement: Supplementary file 2 — pr4c01040_si_002.pdf [file pr4c01040_si_002.pdf]

Affinity purification mass spectrometry on the Orbitrap-Astral mass spectrometer enables high-throughput protein–protein interaction mapping

Lia R. Serrano[1,2], Adrian Pelin[3,4,5], Tabiwang N. Arrey[6], Nicolaie E. Damoc[6], Alicia L. Richards[3,4,5], Yuan Zhou[3,4,5], Trenton M. Peters-Clarke[1,2], Noah M. Lancaster[1,2], Anna Pashkova[6], Gwendolyn M. Jang[3,4,5], Manon Eckhardt[3,4,5], Scott Quarmby,[2,8] Martin Zeller[6], Daniel Hermanson[9], Hamish Stewart[6], Christian Hock[6], Alexander Makarov[6], Vlad Zabrouskov[9], Nevan J. Krogan[3,4,5], Joshua J. Coon[1,2,7,8*], and Danielle L. Swaney[3,4,5*]

[1] Department of Chemistry, University of Wisconsin–Madison, Madison, WI, 53706, USA
[2] Department of Biomolecular Chemistry, University of Wisconsin–Madison, Madison, WI, 53706, USA
[3] J. David Gladstone Institutes, San Francisco 94158 CA, USA
[4] Quantitative Biosciences Institute (QBI), University of California, San Francisco, San Francisco 94158 CA, USA
[5] Department of Cellular and Molecular Pharmacology, University of California San Francisco, San Francisco 94158 CA, USA
[6] Thermo Fisher Scientific GmbH, 28199 Bremen, Germany
[7] National Center for Quantitative Biology of Complex Systems, Madison, WI, 53706, USA
[8] Morgridge Institute for Research, Madison, WI, 53515
[9] Thermo Fisher Scientific, San Jose, CA, 95134, USA

[*]To whom correspondence should be addressed: jcoon@chem.wisc.edu; danielle.swaney@ucsf.edu

Supplementary Data Tables (*Supplementary_Data_Tables.xlsx*)

AEMS_unfiltered. All interactions and AE-MS-based scoring metrics

SAINTq_unfiltered. All interactions and SAINTq-based scoring metrics

MiST_unfiltered. All interactions and MiST-based scoring metrics

AEMS_confident. Confident interactions derived from AE-MS scoring

SAINTq_confident. Confident interactions derived from SAINTq scoring

MiST_confident. Confident interactions derived from MiST scoring

Supplementary Figures

Figure S1. Co-isolation analysis for while HAP1-cell lysate digest separated with a 7-min active gradient and analyzed with Orbitrap Astral MS (2 Th DIA window method)....................................2

Figure S2. Pearson correlation coefficients for all replicates of analyzed baits...................................3

Figure S3. Bait interactions in a network graph depiction for PPIs returned by AE-MS thresholding method…………………………………………………………………………………….…..4

Figure S4. Throughput comparisons between this study and selected protein–protein interaction mapping studies.……………………………………………………………………..……………………5

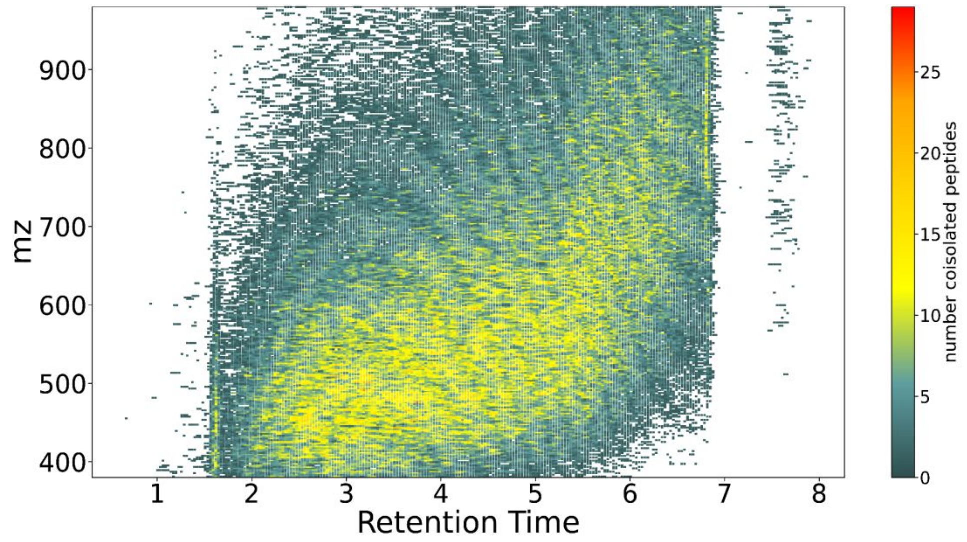

Figure S1. Co-isolation analysis for while HAP1-cell lysate digest separated with a 7-min active gradient and analyzed with Orbitrap Astral MS (2 Th DIA window method). This resulted in a median of five co-isolated precursors per window.

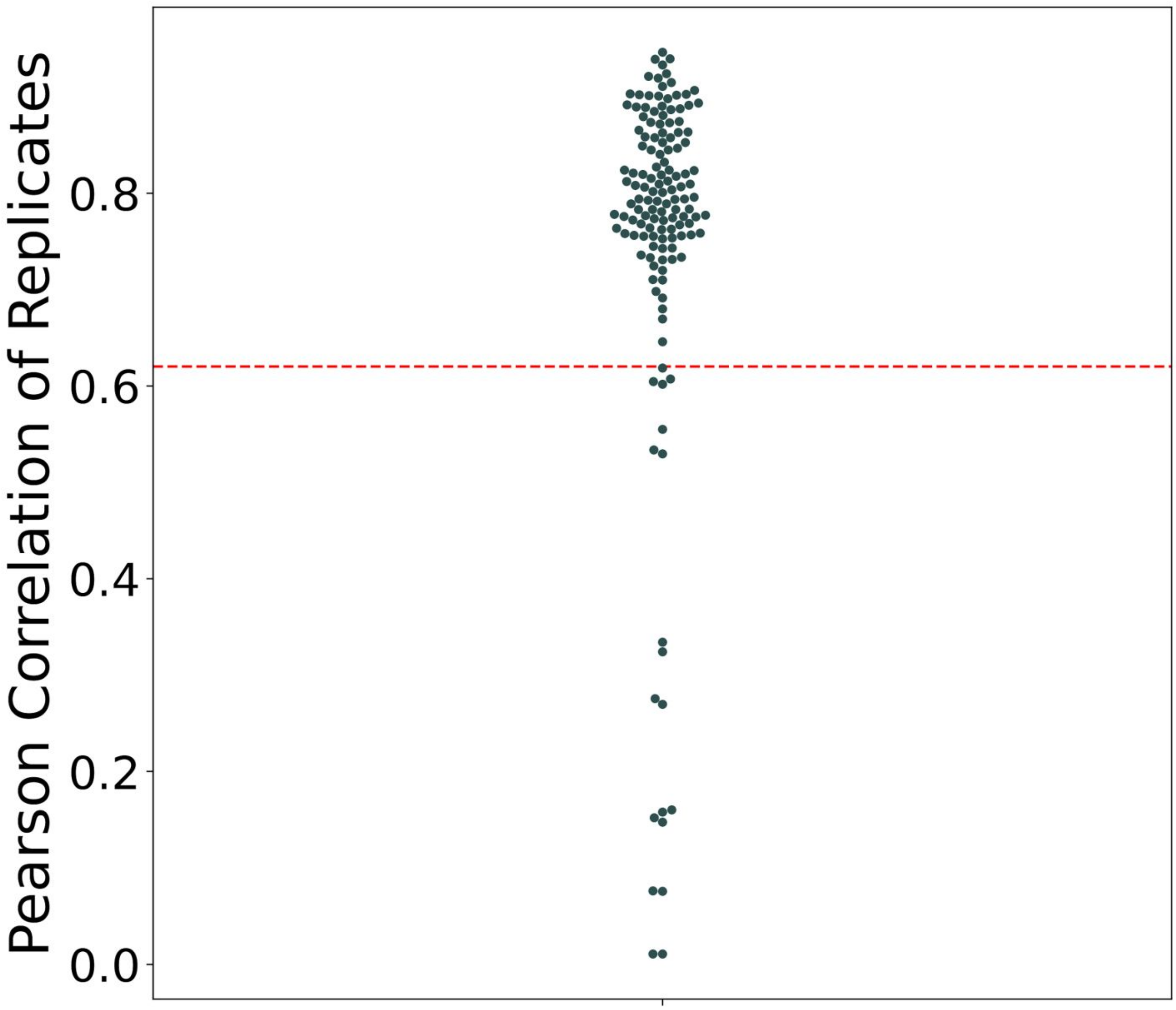

Figure S2. Pearson correlation coefficients for all replicates of analyzed baits. All baits with at least one replicate with a Pearson correlation coefficient below 0.62 (red dotted line) were excluded from further analysis.

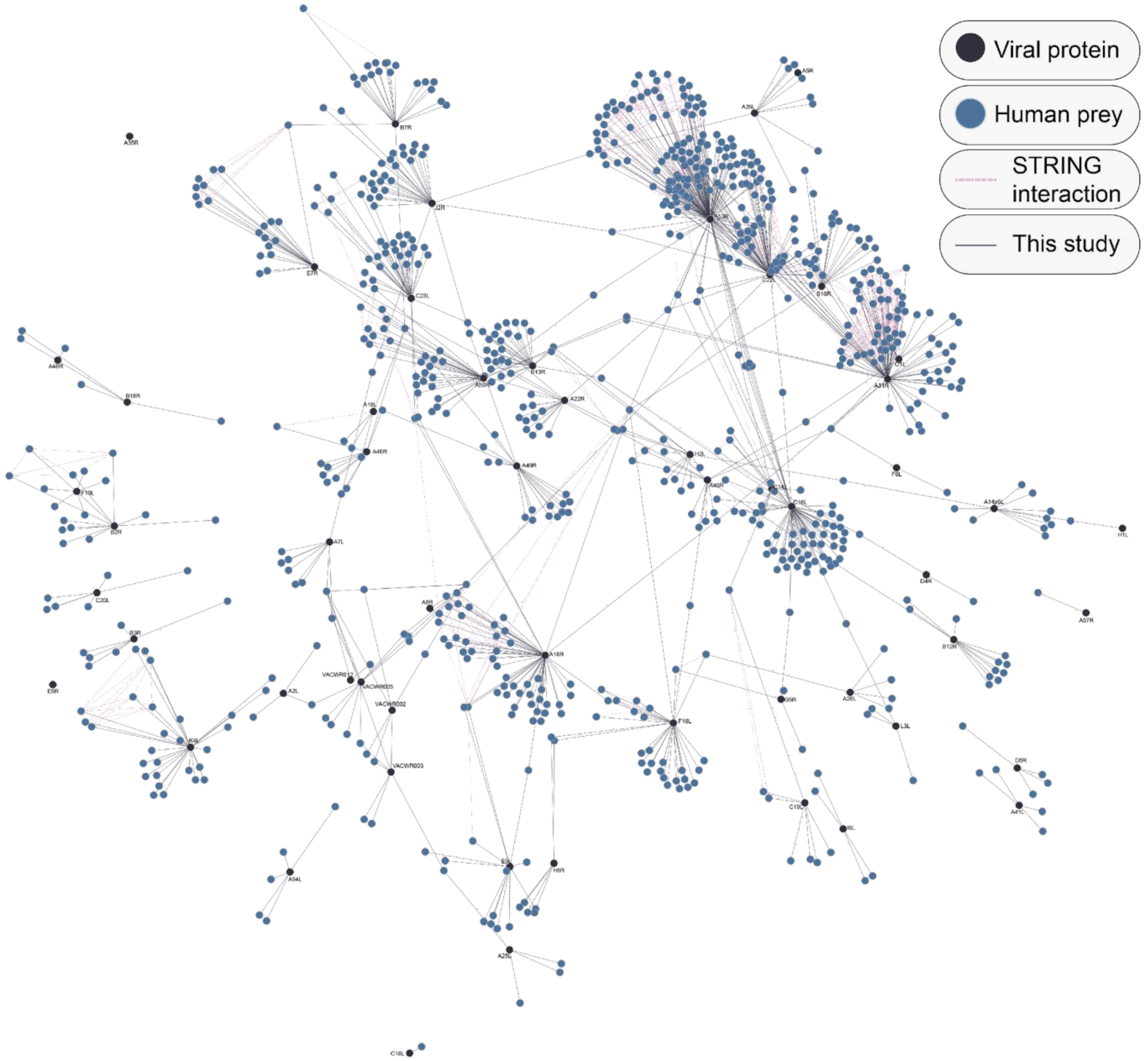

Figure S3. Bait interactions in a network graph depiction for PPIs returned by AE-MS thresholding method. Pink edges denote STRING interactions from the physical links with at least a score of 0.75. The number of human interactor nodes pictured here are consistent with the number of unique genes, from which we measured 998 high-confidence interactors.

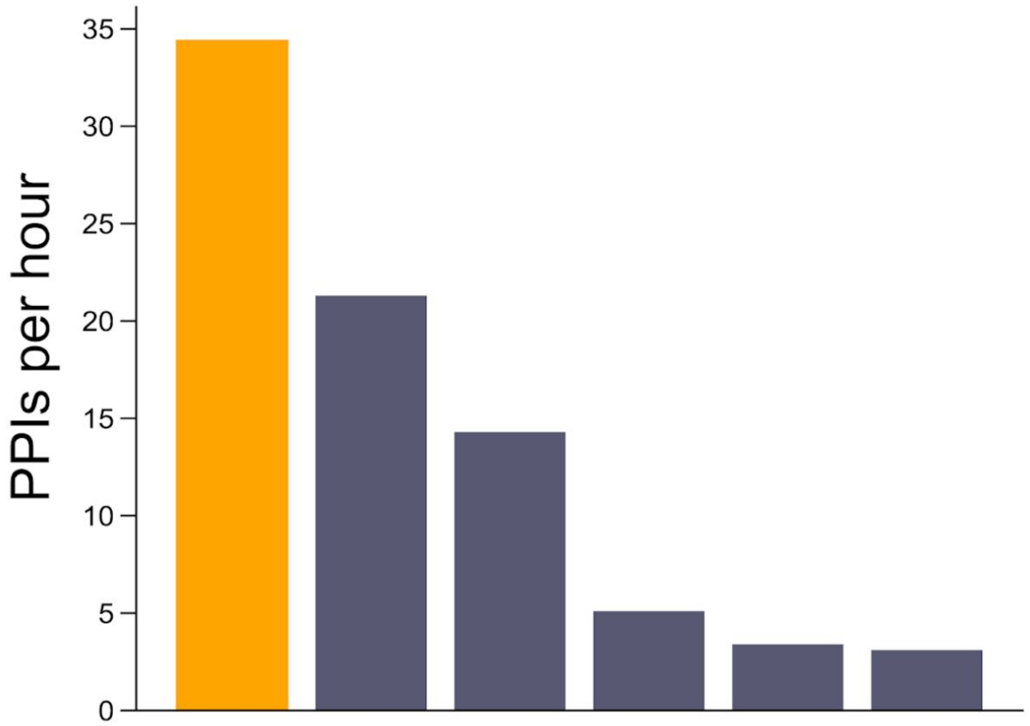

	This Study	Michaelis *et al.* 2023	Bian *et al.* 2020	Kruse *et al.* 2021	Huttlin *et al.* 2021	Gordon *et al.* 2020
Instrument	Orbitrap Astral	timsTOF Pro	Q-Exactive HFX	timsTOF Pro	Q-Exactive	Q-Exactive plus
Total LC-MS Method Duration	8 min	21 min	20 min	60 min	70 min	75 min
Number of Unique Baits	67	6,234	2	57	15,650	26
PPI Scoring Method	AE-MS	AE-MS	SAINTexpress	AE-MS	comPASS-Plus	SAINTexpress MiST

Figure S4. Throughput comparisons between this study and selected protein–protein interaction mapping studies. PPI per hour rates of 34.7(this study), 21.3, 14.3, 5.1 , 3.4 and 3.1 are shown. [1–3,8,10,21,27] The instrument model, total LC-MS method duration, unique number of baits, and PPI scoring strategy are included for each study.
